# “My First 100 Vitrectomies”: Two Years of Collaborative Learning with the Vitreoretinal Fellowship in Switzerland

**DOI:** 10.1055/a-2541-4266

**Published:** 2025-04-16

**Authors:** Giorgio Enrico Bravetti, Emmanouil Blavakis, Iulia Maria Ciotu, Gabriele Thumann

**Affiliations:** Department of Ophthalmology, Geneva University Hospitals, Geneva, Switzerland

**Keywords:** vitreoretinal surgery, rhegmatogenous retinal detachment, Switzerland, fellowship programme, pars-plana vitrectomy, vitreoretinale Chirurgie, rhegmatogene Netzhautablösung, Schweiz, Stipendienprogramm, Pars-plana-Vitrektomie

## Abstract

**Purpose** To evaluate baseline characteristics, intraoperative choices, and surgical outcomes in patients operated by a vitreoretinal (VR) fellow during his first 2 years of fellowship in the Swiss medical system.

**Setting** Longitudinal, monocentric, retrospective study, conducted at the University Hospitals of Geneva.

**Methods** All consecutive cases were included that were operated by the same VR fellow at a tertiary university centre between January 2022 and March 2024. The primary outcome was the surgical success after any surgery. Secondary outcomes were the final best-corrected visual acuity (BCVA) at the final follow-up visit as well as adverse events (AEs).

**Results** One-hundred eyes of 89 patients were included (65 men and 24 women, mean age 61.5 ± 16.1 years, range 22 – 94 years). Out of those 100 vitrectomies, 38.0% were operated by the fellow with the supervisor, who was present in the operating theatre but not sitting at the microscope (group A), 36.0% were operated under direct observation by the supervisor who was sitting at the microscope next to the fellow (group B), and in 26.0% of the eyes with the fellow able to do perform only some parts of the surgery based on his expertise at that time, while the rest was completed by the supervisor (group C). The main type of surgery performed was vitrectomy for rhegmatogenous retinal detachment (52.0%; n = 52) followed by silicone oil removal (13.0%), endophthalmitis (8.0%), epiretinal membrane peeling (8.0%), secondary intraocular lens (7.0%), trauma (5.0%), diabetic haemorrhage or tractional retinal detachment (4.0%), macular hole (2.0%), and vitreous haemorrhage (1%). Out of all these, 58.0% of the operations were classified as emergency (mostly rhegmatogenous retinal detachment (RRD), endophthalmitis and trauma), and 42.0% as elective. Eighty-three operations were standalone vitrectomies, while 17 were combined with cataract surgery. In the group undergoing vitrectomy for RRD, the most frequent operation performed, 48.1% of the operations belong to group A, while the rest was split between groups B and C. Forty eyes (79.6%) were classified as recent and uncomplicated RRD, and 12 (23.1%) as long-standing or complicated RRD. Most of the RRD cases were macula-on (59.6%; n = 31) and phakic (69.2%; n = 36). In order to manage RRD, perfluorocarbon liquid was used in 40.4% of the eyes, and retinotomy was performed in 26.9% of the cases. Thirty-four patients (65.4%) required a 360° laser retinopexy and ab-externo cryocoagulation of the retinal tears. The most often used endotamponade for RRD surgery was 14% C3F8 gas (42.3%; n = 22) followed by 20% SF6 gas (26.9%; n = 14), heavy silicone oil (13.5%; n = 7); 1000 cSt silicone oil (9.7%; n = 5), and 5000 cSt silicone oil (3.9%; n = 2). Out of all operations, mean BCVA improved significantly during follow-up (0.32 ± 0.34 decimals at baseline vs. 0.47 ± 0.30 decimals at the final follow-up; p < 0.05). Four eyes (4.0%) were classified as failures and required a subsequent operation, mostly for recurrent RRD. The most frequent AE was post-operative cystoid macular oedema, which occurred in 4.0% of the eyes, and cataract development, which occurred in almost all patients who were phakic at the time of surgery. One patient experienced a lens touch during the surgery for RRD and had developed a white cataract at post-operative follow-up. Average follow-up time was 5.5 ± 5.4 months (range 1 – 24 months).

**Conclusions** This study provides a comprehensive analysis of the surgical outcomes and intraoperative experiences of a vitreoretinal fellow during their initial 2-year fellowship in Switzerland. The majority of operations, notably for rhegmatogenous retinal detachment, gave successful outcomes with significant improvements in visual acuity observed post-operatively. The collaborative approach between the fellow and the supervisors varying from direct observation to independent performance showcases a structured training environment. Despite encountering some adverse events, our findings underscore the effectiveness of the fellowship programme in imparting valuable surgical skills and achieving favourable patient outcomes in vitreoretinal surgery.

## Zusammenfassung

**Ziel** Bewertung der Ausgangsmerkmale, intraoperativen Entscheidungen und chirurgischen Ergebnisse bei Patienten, die von einem vitreoretinalen (VR) Fellow während seiner ersten zwei Jahre der Fellowship im schweizerischen Gesundheitssystem operiert wurden.

**Setting** Längsschnittliche, monozentrische, retrospektive Studie, durchgeführt an den Universitätsspitälern Genf.

**Methoden** Eingeschlossen wurden alle aufeinanderfolgenden Fälle, die von demselben VR Fellow in einem tertiären universitären Zentrum zwischen Januar 2022 und März 2024 operiert wurden. Das primäre Ziel war der chirurgische Erfolg nach einer Operation. Sekundäre Endpunkte waren die beste korrigierte Sehschärfe (BCVA) beim letzten Nachsorgetermin sowie unerwünschte Ereignisse (AEs).

**Ergebnisse** Es wurden 100 Augen von 89 Patienten eingeschlossen (65 Männer und 24 Frauen, Durchschnittsalter 61,5 ± 16,1 Jahre, Bereich 22 – 94 Jahre). Von diesen 100 Vitrektomien wurden 38,0% vom Fellow unter Aufsicht durchgeführt, wobei der Supervisor im Operationssaal anwesend, aber nicht am Mikroskop tätig war (Gruppe A). In 36,0% der Fälle wurde die Operation unter direkter Aufsicht des Supervisors durchgeführt, der neben dem Fellow am Mikroskop saß (Gruppe B). In 26,0% der Fälle führte der Fellow nur einige Teile der Operation entsprechend seiner aktuellen Expertise durch, während der Supervisor den Rest übernahm (Gruppe C). Die häufigste Operationsindikation war die Vitrektomie bei rhegmatogener Netzhautablösung (52,0%; n = 52), gefolgt von der Entfernung von Silikonöl (13,0%), Endophthalmitis (8,0%), epiretinaler Membran-Peeling (8,0%), sekundäre intraokulare Linse (7,0%), Trauma (5,0%), diabetische Blutung oder traktive Netzhautablösung (4,0%), Makulaforamen (2,0%) und Glaskörperblutung (1%). Von diesen Operationen wurden 58,0% als Notfälle eingestuft (meist rhegmatogene Netzhautablösung, Endophthalmitis und Trauma) und 42,0% als elektive Eingriffe. 83 Operationen waren reine Vitrektomien, während 17 mit einer Kataraktoperation kombiniert wurden. In der Gruppe mit rhegmatogener Netzhautablösung (RRD), der häufigsten Operationsindikation, entfielen 48,1% der Eingriffe auf Gruppe A, während die restlichen auf Gruppen B und C verteilt waren. Vierzig Augen (79,6%) wurden als frische und unkomplizierte RRD klassifiziert, 12 (23,1%) als langbestehende oder komplizierte RRD. Die meisten RRD-Fälle waren makulaanliegend (59,6%; n = 31) und phakisch (69,2%; n = 36). Zur Behandlung der RRD wurde in 40,4% der Fälle Perfluorcarbon-Flüssigkeit verwendet, und in 26,9% der Fälle wurde eine Retinotomie durchgeführt. 34 Patienten (65,4%) erhielten eine 360°-Laserretinopexie und eine ab-externo Kryokoagulation der Netzhautrisse. Das am häufigsten verwendete Endotamponade-Material für die RRD-Operation war 14% C3F8-Gas (42,3%; n = 22), gefolgt von 20% SF6-Gas (26,9%; n = 14), schwerem Silikonöl (13,5%; n = 7), 1000 cSt Silikonöl (9,7%; n = 5) und 5000 cSt Silikonöl (3,9%; n = 2). Über alle Operationen hinweg verbesserte sich die durchschnittliche BCVA signifikant im Verlauf der Nachsorge (0,32 ± 0,34 Dezimalwerte zu Beginn vs. 0,47 ± 0,30 Dezimalwerte am letzten Nachsorgetermin; p < 0,05). Vier Augen (4,0%) wurden als Misserfolge eingestuft und benötigten eine erneute Operation, meist aufgrund eines rezidivierenden RRD. Das häufigste unerwünschte Ereignis war das postoperative zystoide Makulaödem, das in 4,0% der Fälle auftrat, sowie die Kataraktentwicklung, die bei fast allen phaken Patienten nach der Operation beobachtet wurde. Ein Patient erlitt während der Operation einer RRD eine Linsenberührung und entwickelte postoperativ eine weiße Katarakt. Die durchschnittliche Nachbeobachtungszeit betrug 5,5 ± 5,4 Monate (Bereich 1 – 24 Monate).

**Schlussfolgerungen** Diese Studie liefert eine umfassende Analyse der chirurgischen Ergebnisse und intraoperativen Erfahrungen eines vitreoretinalen Fellows während der ersten zwei Jahre seiner Fellowship in der Schweiz. Die Mehrheit der Operationen, insbesondere bei rhegmatogener Netzhautablösung, zeigte erfolgreiche Ergebnisse mit einer signifikanten Verbesserung der Sehschärfe nach der Operation. Der kollaborative Ansatz zwischen dem Fellow und den Supervisors – von direkter Beobachtung bis hin zur eigenständigen Durchführung – veranschaulicht eine strukturierte Ausbildung. Trotz einiger unerwünschter Ereignisse unterstreichen unsere Ergebnisse die Effektivität des Fellowship-Programms bei der Vermittlung wertvoller chirurgischer Fähigkeiten und der Erzielung günstiger Patientenergebnisse in der vitreoretinalen Chirurgie.

## Introduction

The journey to becoming a vitreoretinal (VR) surgeon is rigorous, involving extensive training and progressive exposure to complex ocular surgeries 1 . Fellowship programmes play a pivotal role in this process by providing aspiring surgeons with hands-on experience under expert supervision. However, during this period, fellows are often tasked with performing surgeries independently or with varying levels of supervision, which raises questions regarding surgical outcomes, patient safety, and the fellowʼs learning curve 2 . To better understand the nuances of this transition from trainee to competent surgeon, it is important to evaluate the performance and outcomes associated with surgeries performed by fellows early in their training 3 , 4 .

The Swiss medical training system, known for its high standards of care, provides a structured environment for surgical fellows. Fellows typically start with limited responsibilities, gradually progressing to more complex procedures as they gain experience 5 . However, with an estimated population of fewer than 9 million people, Switzerland is a relatively small country compared to its neighbours such as Italy, France, and Germany. For instance, if we assume an incidence of 1.4 in 10,000 for rhegmatogenous retinal detachment (RRD) in Europe, this would result in fewer than 1,300 cases per year in Switzerland 6 . This raises the following question: Is this enough to adequately train the next generation of Swiss VR surgeons?

The purpose of this study was to evaluate baseline characteristics, intraoperative choices, and surgical outcomes of patients operated by a single VR fellow during his first 2 years of fellowship in the Swiss medical system. We believe that such a longitudinal analysis is essential for assessing competency development in surgical fellows and the associated patient outcomes, especially for VR surgery that is a specialised field requiring extensive training and progressive acquisition of surgical skills.

## Methods

### Study design

The present study was a longitudinal, monocentric, retrospective study conducted at a tertiary university centre (University Hospitals of Geneva). Written informed consent was obtained from all patients included in the study. The study complies with the tenets of the Declaration of Helsinki and was approved by the local ethical committee (Swissethics, study number 2024-01359).

### Study population

Results of all subsequent eyes presenting vitreoretinal diseases operated by standard 23- or 25-gauge pars-plana vitrectomy by the same VR fellow (G. E. B.), between January 2022 and March 2024 were analysed.

### Baseline measurements

For each patient, the following demographics and clinical characteristics were recorded: age, gender, ethnicity, diagnosis and ophthalmic/surgical history, best corrected visual acuity (BCVA), and axial length. When possible, tomography (SD-OCT [Spectralis OCT, Heidelberg Engineering AG, Germany]) and ultra-widefield fundus photography (Optos California, Nikon Co. Ltd, Japan) were performed based on the vitreous and retinal status a spectral-domain optical coherence.

### Outcomes and measures

The primary outcome was the surgical success after any surgery. Loss of light perception, serious irreversible complications, retinal re-detachment in case of primary RRD, and any subsequent surgical interventions were considered surgical failure. Secondary outcomes were the final BCVA at the final follow-up visit and the adverse events (AEs) rate. Safety endpoints included the rate of intraoperative complications and post-operative AEs during the entire follow-up period.

### Statistical analysis

Descriptive statistics included mean and standard deviation (SD) for normally distributed variables, and median and interquartile range (IQR) for non-normally distributed variables. All tests were two-tailed, and a p-value of less than 0.05 was considered statistically significant. Statistical analyses were performed with commercially available software (Stata version 13.1; StataCorp, College Station, TX, USA).

## Results

### Baseline characteristics of study population and type of surgeries

One-hundred eyes in 89 patients were included (65 men and 24 women, mean age 61.5 ± 16.1 years, range 22 – 94 years). Out of those 100 pars-plana vitrectomies, 38.0% were operated by the fellow with the supervisor being present in the operating theatre but not sitting at the microscope and not directly assisting (group A), 36.0% were operated under the direct observation of the supervisor who was sitting at the microscope next to the fellow providing close guidance (group B), and in 26.0% of the eyes, the fellow was able to perform only some parts of the surgery based on his competence at that time, while the rest was completed by the supervisor (group C).

The main type of surgery performed was vitrectomy for rhegmatogenous retinal detachment (52.0%; n = 52) followed by silicone oil removal (13.0%), endophthalmitis (8.0%), epiretinal membrane peeling (8.0%), secondary intraocular lens (7.0%), trauma (5.0%), diabetic haemorrhage or tractional retinal detachment (4.0%), full-thickness macular hole (2.0%), and vitreous haemorrhage (1%). Out of all surgeries, 58.0% were classified as emergencies (mostly RRD, endophthalmitis and trauma) and 42.0% as elective. Eighty-three surgeries were standalone vitrectomies, while 17 were combined with cataract surgery. Baseline characteristics of the study patients are summarised in Table 1 .

**Table:** **Table 1** Baseline demographics and clinical characteristics of study population and type of surgery performed.

Demographic and clinical data	Mean ± SD (%)
Age (years)	61.5 ± 16.1
Range	22 – 94
Male gender	65 (65.0%)
Ethnicity	
White	92 (92.0%)
Black	6 (6.0%)
South or East Asian	2 (2.0%)
Bilateral cases	11 (11.0%)
Diagnosis	
RRD	52 (52.0%)
SO removal	13 (13.0%)
Exogenous endophthalmitis	8 (8.0%)
ERM peeling	8 (8.0%)
Secondary IOL	7 (7.0%)
Trauma	5 (5.0%)
Diabetic TRD	4 (4.0%)
FTMH	2 (2.0%)
Vitreous haemorrhage	1 (1.0%)
Emergency surgeries	58 (58.0%)
Elective surgeries	42 (42.0%)
Standalone PPV	83 (83.0%)
Combined surgeries	17 (17.0%)
Group A	38 (38.0%)
Group B	36 (36.0%)
Group C	26 (26.0%)
Baseline BCVA (decimal)	0.32 ± 0.34
RRD: rhegmatogenous retinal detachment; SO: silicone oil; ERM: epiretinal membrane; IOL: intraocular lens; TRD: tractional retinal detachment; FTMH: full thickness macula hole; PPV: pars-plana vitrectomy; BCVA: best-corrected visual acuity	

### Characteristics of the subgroup of vitrectomies for rhegmatogenous retinal detachment

In the group of vitrectomy for RRD, the most frequent surgery performed, 48.1% of the surgeries belonged to group A, while the rest was split between groups B and C. Forty eyes (79.6%) were classified as recent and uncomplicated RRD, while 12 (23.1%) were classified as long-standing or complicated RRD. Most of the RRD cases were macula-on (59.6%; n = 31) and phakic (69.2%; n = 36). In order to manage RRD, perfluorocarbon liquid was used in 40.4% of the eyes, and retinotomy was performed in 26.9% of the cases. Thirty-four patients (65.4%) required a 360° laser retinopexy and ab-externo cryocoagulation of the retinal tears. The most used endotamponade for RRD surgery was 14% C3F8 gas (42.3%; n = 22), followed by 20% SF6 gas (26.9%; n = 14), heavy silicone oil (13.5%; n = 7); 1,000 cSt silicone oil (9.7%; n = 5), and 5,000 cSt silicone oil (3.9%; n = 2). Characteristics of the RRD group are summarised in Table 2 .

**Table:** **Table 2** Clinical characteristics of the rhegmatogenous retinal detachment subgroup.

Clinical data	Mean ± SD (%)
Type of RRD	
Recent and uncomplicated	40 (79.6%)
Long-standing or complicated	12 (23.1%)
Macula status	31 (59.6%)
Macula-ON Macula-OFF	21 (40.4)
Lens status	
Phakic	36 (69.2%)
Pseudophakic	16 (30.8%)
Endotamponade	
14% C _3_ F _8_ gas	22 (42.3%)
20% SF _6_ gas	14 (26.9%)
Heavy SO (Densiron)	7 (13.5%)
1,000 cSt SO	5 (9.7%)
5,000 cSt SO	2 (3.9%)
RRD: rhegmatogenous retinal detachment; SO: silicone oil	

### Primary outcome: surgical success

Out of all eyes, surgical success was 96% with only four eyes (4.0%) classified as failure requiring a subsequent surgery. All of those belonged to the RRD group and required subsequent surgical intervention because of a recurrent detachment.

### Secondary outcomes

Out of all surgeries, mean BCVA improved significantly during follow-up (0.32 ± 0.34 decimals at baseline vs. 0.47 ± 0.30 decimals at final follow-up; p < 0.05). No intraoperative complications were noted among the studied cohort. None of the combined procedures were associated with posterior capsule rupture or the need for anterior vitrectomy. All pars-plana vitrectomies were uneventful and without peri-operative complications. The most frequent AE was post-operative cystoid macular oedema that occurred in 4.0% of the eyes, and cataract development that occurred in almost all patients who were phakic at the time of surgery. One patient operated using 23-gauge vitrectomy experienced a lens touch during the surgery for RRD and developed a white cataract at post-operative follow-up. Average follow-up time was 5.5 ± 5.4 months (range 1 – 24 months). Primary and secondary outcomes are summarised in Table 3 .

**Table:** **Table 3** Primary and secondary outcomes.

Outcome	Mean ± SD (%)
Surgical success	96 (96.0%)
Surgical failure	4 (4.0%)
BCVA at final follow-up (decimals)	0.47 ± 0.30
Post-operative AEs	
Re-detachment	4 (4.0%)
CMO	4 (4.0%)
Lens touch with subsequent white cataract	1 (1.0%)
BCVA: best corrected visual acuity; AE: adverse events; CMO: cystoid macular oedema	

## Discussion

The present study aimed to assess the surgical outcomes and intraoperative experiences of a single VR fellow during his first two years of training in the Swiss medical training system. Most surgeries, particularly those for rhegmatogenous retinal detachment, resulted in successful outcomes with marked post-operative improvements in visual acuity. The strength of this study is that the training environment was structured with the fellow and the supervisors collaborating in various capacities. At the time, the VR fellow was the only VR surgeon in training within the fellowship programme and participated in the on-call retinal surgery rotation, which included three retinal surgeons in total, resulting in being on call for more than 120 days per year. This allowed the recruitment of 58 emergency cases (including RRD, endophthalmitis and trauma cases) where a pars-plana vitrectomy was required. As a result, the fellow performed 100 vitrectomies over two years of training, likely because supervisors tend to be more comfortable allowing fellows to operate in emergencies rather than elective cases. A study performed in the United Kingdom (UK) showed that it was safe for experienced VR fellows to perform surgery during weekends and holidays, supervised or unsupervised, as they had similar success rates and visual outcomes to more experienced surgeons 2 .

There are no official requirements for the completion of a VR surgery programme in Switzerland, while in the United States (US), the Association of University Professors of Ophthalmology (AUPO) guidelines for VR fellowships require that the fellow performs or assists at a minimum of 40 scleral buckles and 100 vitrectomies 1 . Although performing 40 scleral buckles is almost impossible during training in Switzerland, as this procedure is nowadays rarely performed, the requirements regarding vitrectomies were achieved in a two-year period in our study. However, fellows in the US most commonly perform 300 to 400 vitrectomies 1 . In the UK, the British & Eire Association of Vitreoretinal Surgeons (BEAVRS) requires the completion of at least 250 vitrectomies to become a consultant VR surgeon, a number that is probably difficult to achieved in a 2-year Swiss fellowship programme due to the low case volume 7 . In a survey that assessed the experience of VR fellows in the UK, an average of 351 vitrectomies were performed during the fellowship 8 . According to the same survey, most cases were vitrectomies for retinal detachment as in our study, but the fellows also performed a much greater number of vitrectomies for epiretinal membranes peels (76 in the UK versus 8 in this study) and diabetic delamination (16 in the UK versus 4 in this study) 8 .

The success rate of vitrectomy for RRD in this study was 96%. This is much higher than the 70% success rate of less experienced surgeons reported by Dugas et al. in a French study 9 . In a similar study of Radeck et al. performed in Germany, the success rate of inexperienced surgeons was about 80% and was stabilised at more than 90% only after performing 200 vitrectomies 10 . Keller et al. also showed that surgeons in training saw a recurrent retinal detachment rate of 14.7% during their learning curve 11 . On the contrary, the results of our study were more similar to those of Mazinani et al., who reported a success rate of 90%; interestingly, this was not related to the number of vitreoretinal procedures performed 3 .

The relatively low re-detachment rate in this study could be explained by the fact that the fellow in training was directly supervised in most of surgeries, as only 38% of surgeries were performed with the supervisor attending not sitting at the microscope. As shown by Masson et al., surgeons in training can reach similar visual outcomes and complication rates than experienced surgeons under proper supervision 4 . In addition, all patients were operated under deep sedation or general anaesthesia according to the departmentʼs usual practice. This probably allows better communication in the operating room between the fellow and the supervisor and facilitates guidance of the surgeon in training.

The conservative approach of the fellow with the frequent use of 14% C _3_ F _8_ gas as endotamponade (used in 42.3% of RRD) and the common application of 360° laser retinopexy and ab-externo cryocoagulation (in 65.4% of RRD) may have also contributed to the high success rate. It is possible that a more experienced surgeon would more frequently use a 20% SF _6_ gas versus 14% C _3_ F _8_ gas and would more often prefer gas over silicon oil as endotamponade compared to a young surgeon in training.

Out of 100 vitrectomies, the fellow also took on some cases considered more challenging by young surgeons such us ocular trauma requiring vitrectomy (5%), secondary intraocular lens implantation (7%), and diabetic tractional retinal detachment (4%). Due to their small number, the success rate of those procedures cannot be evaluated. However, it is important for fellows to perform such types of surgeries as well, as it is has been shown that, in diabetic tractional retinal detachment for example, the anatomical success rates increases from 85% to 96% from the first to second year of training 12 .

Several additional reasons could contribute to the high success rate observed in this study. The fellow had the opportunity to assist many VR surgeries during his residency before starting his hands-on experience. Moreover, he was also trained in cataract and glaucoma surgery before the VR fellowship. While Thomsen et al. demonstrated that surgical skills gained during cataract surgery training donʼt necessarily enhance vitreoretinal surgery performance, we believe that any opportunity to perform intraocular surgery can improve a traineeʼs dexterity and boost their confidence 13 . Watching multiple surgical videos is also known to promote surgical planning, add to skills improvement, and facilitate situational awareness during vitreoretinal surgery 14 . The recent availability of three-dimensional (3D) videos could further enhance the teaching efficiency of training in intraocular surgery 15 . The importance of the availability of mentors that are committed in training, dedicating time in supervising fellows, and giving them the opportunity to operate should also not be underestimated 16 . Although not used in this study, a 3D heads-up display has been shown to promote surgical training, and we believe that it could be extremely helpful in fellowship programmes 17 , 18 .

Finally, another tool that could be used for the training of fellows in countries with low patient volumes like Switzerland is a surgery simulator. The fellow in this study completed the cataract and vitreoretinal surgery training programme of the Eyesi surgical simulator (VRMagic, Haag-Streit, Switzerland) prior to the beginning of the fellowship. Surgical simulators are considered one of the best training tools for inexperienced surgeons and the skills acquired while using them are thought to be directly transferred to the operating theatre 19 . They can also be used to evaluate the surgical competence of fellows before operating on patients and they can probably help identify surgical steps where more training is needed 20 . In low volume fellowship programmes, they can also be used by the fellows for “warming up” before surgery 21 . Unfortunately, their use is limited by high costs, which make surgical simulators unavailable in most centres, and by the lack of time of fellows, who are unable to take full advantage of them even when accessible 19 , 22 .

The present study has several limitations. First, it was not a prospective randomised controlled comparative study and there was no control group. The fact that all cases were operated by the same VR fellow at a single tertiary university centre, may be considered as both a limitation or a strength. This led to some degree of standardisation in surgical techniques and clinical decisions; however, some aspects of patient management inherently depend on the surgeonsʼ own experience and judgement. As a result, the findings likely reflect only this particular fellowʼs experience and may not be generalisable. Moreover, in this study, vitrectomy was the sole treatment performed for RRD, as it is the gold standard at the institution. However, alternative procedures such as scleral buckle, pneumatic retinopexy (PnR), or viscopexy could have been considered for many of the cases. While PnR is an established and effective method for treating certain types of RRD, it is not yet widely practiced in Switzerland. The future adoption of this technique could potentially decrease the number of vitrectomies performed by fellows 23 . That said, the limited use of PnR by fellows is not unique to Switzerland, as a 2019 study revealed that in the US, as of 2016, the median number of PnR procedures performed by VR fellows per year was just 7 24 . Finally, another limitation of our study is that it was conducted in a predominantly homogenous (western) population.

In conclusion, this study offers an in-depth assessment of the surgical outcomes and intraoperative experiences of a vitreoretinal fellow during his first two years of fellowship in Switzerland. Most surgeries, particularly those for rhegmatogenous retinal detachment, resulted in successful outcomes with marked post-operative improvements in visual acuity. The training environment was structured with the fellow and the supervisors collaborating in various capacities ranging from direct supervision to independent practice. Although some complications were encountered, our findings highlight the programmeʼs effectiveness in developing essential surgical skills and delivering positive patient outcomes in vitreoretinal surgery.
